# Organic Poisoning: A Case of Snake Bite with Some Observations on the Same

**Published:** 1870-02

**Authors:** O. F. Potter

**Affiliations:** St. Louis, Mo., Prof. Mat. Med. and Medical Botany, St. Louis College of Pharmacy, and Corresponding Member of the Muskingum County Medical Society, Zanesville, Ohio


ART. II.—*Organic Poisoning: A case of Snake Bite with some observations on the same, by* O. F. POTTER, M. D., *St. Louis, Mo., Prof. Mat. Med .and Medical Botany, St Louis College of Pharmacy, and Corresponding Member of the Muskingum County Medical Society, Zanesville, Ohio.*

Communicated to the Muskingum County Medical Society, and read at its session 6th January, 1870.

*Also, a letter to* Prof. POTTER *on Organized Poisons, their modus operandi, therapeutic and remedial management, from a physical basis of life. By* Z. C. MCELROY, M. D., *Zanesville, O., President Muskingum County Medical Society.*

FELLOWS OF THE SOCIETY:

In compliance with a promise made to my kind friend, your worthy President, I take the liberty of laying the following case before you. I can only plead ill health, and pressing professional engagements as my apology for not having sooner given some return for the honor you have so kindly conferred in electing me a corresponding member of your society; which I take more as evidence of the good will of my friends, than from any merit of my own. While submitting the following case, I ask your indulgence; trusting at some future time to offer something more worthy your consideration.

During an excursion in the summer of 1862, to visit some friends up the country, I stopped on the road side, near a harvest field, to converse with the owner. Very soon one of the harvest hands came running up to where we were talking, and told us he had been bitten by a rattlesnake, on the inside of one of his forearms, near the middle, and knowing me to be a physician, appealed to me for help. One of the other hands soon came up with the snake, which proved to be a prairie rattlesnake, of large size. I had no medicines at hand, neither was there any at the house of the proprietor. But, nevertheless, something must be done. So, taking a pocket lancet, the arm was freely scarified at the points wounded by the serpent. The man was directed to suck the blood with his lips, and spit it out, which he did vigorously. A dressing of moistened clay was applied, and loosely confined by a handkerchief; as the arm, by this time had become somewhat painful, and commenced swelling. I

now proposed, as the next best thing I could do, to take the man in my buggy to the village, some eight or ten miles distant, to obtain means for further treatment. Before starting, however, he complained that a stupor was creeping over him. And now comes the point of interest in the case, and to which your attention is directed. Recollecting that in opium, and in other kinds of poisoning, that active exercise aided much in throwing off their effects, I proposed to him to run rapidly a short distance from us, and return. He did as I requested, returning in a profuse perspiration. Getting in the buggy we now started for the village. In a little time he complained again that the stupor was coming over him again. I had him get out and take hold of the back of the carriage, and run until he was very much exhausted, and sweating profusely. This was repeated as often as he complained of the stupor returning; so that when we reached the village, he had run more than half the way, and his clothing was completely saturated with sweat. In the village, I gave him the usual whiskey treatment, with ammonia; but was surprised to find that a small quantity, comparatively, of whisky, produced intoxication; while in previous cases I had seen that large quantities could be taken without producing such effect. To conclude, the case readily yielded to treatment, and in a very short time he made a good recovery, with few bad, and no alarming symptoms.

The points of the case can be summed up as follows:

1st. The patient was severely bitten by a rattlesnake;

2d. He suffered from, and was completely affected by the poison;

3d. He had no treatment but the drawing of the blood, and the the clay poultice, for some two or three hours, except the violent exercise; and,

4th. When the whisky was administered he, usually borne in large quantities, intoxication readily supervened, showing to my mind, an elimination of the virus; and,

5th. A rapid and favorable recovery.

I am convinced that from the rapid and violent circulation of the blood by the exercise, that the poison was prevented fiom accumulating, or concentrating in the vital parts of the brain, and was carried out of the system by the exciting organs, as the skin, kidneys, &c.; and by the rapidity of their elimination, prevented that destruction, or deterioration of vital action, which would so speedily terminate life, and tended, in a great degree, to the final recovery of the patient, and the comparatively mild action of the poison in his system.

Since the above case was observed by me, I noticed in the Bombay *Medical Gazette*, 1867 or 1868, an account of a British officer in India, who was bitten by a venomous serpent, and who had to run some three miles to his station for treatment, and to the violent exercise in running, profuse perspiration which followed, it was supposed he owed his life, he having recovered with but few bad symptoms, from what is, in that country, usually considered a fatal poison. It is also stated that when the jugglers, or any of the natives of India are bitten by venomous snakes they dance violently, until they drop down from sheer exhaustion, and that those rarely die who carry out fairly this treatment.

In connection with this case, permit me to call your attention to the following extract from a communication from my esteemed friend, Dr. J. E. Nield, of Melbourne, Australia, dated 29th May, 1869:

"* * * On the subject of snake bite, there have been some interesting experiments of late. Of them you have no doubt heard, but you have evidently not received those numbers of the journal containing an account of Professor Halford's experiments, on the subject of ammonia injection, (into the veins), in Snake poisoning. As he is about to read a paper before the Medical Society of this Colony, detailing the history of his investigations on this very interesting and important subject; and as it will be published in the *Australian Medical Journal*, I shall have much pleasure in drawing your attention to it. Briefly stated, it consists in the direct introduction into the blood of liquor ammoniæ, as an efficient remedy in snake poisoning. The old fear of injury resulting from the access of air into the current of the circulation is demonstrated to be groundless.

Professor Halford and myself have been experimenting largely on dogs. We have injected, not only into the veins, but into the cavities of the head—the thoracic viscera having been exposed—and there has not only not been the least injury occasioned, but positive benefit, the heart's action being augmented after each injection. We are now engaged in testing the effect of this agent in poisoning by other nerve influencing agents, and so far, the results have been very remarkable."

"The communication from Professor Halford, including the several accounts of Messrs. A. Beckett, Arnold and Wooldiidge, of the recent case of snake poisoning at Brighton, is the first which has been recorded in this Journal, corroborative of the conclusions to which Professor Halford was led, by his experiments on dogs, that the injection of Liquor Ammoniæ into the veins, offers the most immediate and certain means of counteracting the effects of this toxic agent. Nothing could be more conclusive than the testimony of these three gentlemen, and if one case might establish the value of a remedy, there should be no hesitation in pronouncing the ammonia injection treatment as the specific in snake bite. Four other cases have occurred, three of them in this colony, the fourth in New South Wales, and each of them corroborate the belief that Professor Halford's discovery is one of great value.—*Australian Medical Journal.*

*To the Editor of the Australian Medical Journal.*

Sir:—I forward you some particulars of the case of Snake poisoning (the description of Snake being the *Hoplocephalus Superbus*, closely allied to the Tiger Snake), as I have obtained them through the kindness of Messrs. Arnold, A'Becket and Wooldridge, Surgeons in attendance.

Mr. Arnold writes thus: At about eleven o'clock, Monday morning, November 30th 1868, Mr. John Brown, Station Master at Elsternwick, was bitten on the third finger of the right hand. Two punctures were clearly visibly, from which the blood flowed. Symptoms: pain in the finger, wild appearance of countenance, stiffness of legs. Applied a ligature above the bite, employed suction to the wound, and took sixpenny worth of brandy. 1 P. M., felt drowsy, and unable to attend to his duties. As he did not feel alarmed, his friends had great difficulty in persuading him to take 1:17 train to town for advice. Feeling somewhat better at Balaclava, (four miles journey), he declined to proceed further. Whilst conversing with Mr. McPherson, the latter noticed his manner resembling that of a person laboring under the effects of drink

—speech affected, drowsiness. He was then brought almost against his will, to my residence, five minutes' walk; about half-way he complained of being very weak. On his arrival, he was completely prostrated, and shortly after paralysis of the lower extremities set in, with indistinct vision, sluggish dilated pupils, weak pulse, cold perspiration and vomiting. Treatment—excision of bitten part, firmer ligature. In a short time symptoms of coma commenced; when roused with difficulty, he had great trouble in recognizing his wife, or any particular friend, considerable swelling noticed about upper lip. Treatment: gave brandy, in all about six ounces, swallowed with great difficulty, Sal Volatile, half an ounce, in divided doses; Mustard poultices to the head and feet; Galvanism, which greatly roused the patient; the moment it was suspended the comatose symptoms returned; at the same time it was evident this artificial restorative would fail, coma becoming deeper and deeper. At 5:30 P. M., Mr. Wooldridge, and Professor Halford came to our assistance.

Mr. A. Becket writes: * * * about this time we determined upon injecting Ammonia, as we considered the case to be desperate. An attempt to walk the patient about the room, so far from being attended with any good results, seemed to prostaate him utterly; complete paralysis of the lower extremities, having now succeeded to the nervous weakness and impaired sensation in the legs. The effect of the operation, (injection of Ammonia), performed by Professor Halford * was wonderful, the patient reviving at once, conciousness returning, the pulse becoming full; the pupil acting readily to the stimulus of light, The relief of the symptoms was persistent and the patient, though experiencing extreme debility, has gradually advanced to convalesence. I will end the account of this highly interesting case by stating my belief that the injection of Ammonia saved the man's life.

*The modus operandi, therapeutic and remedial agencies of organised poisons.*

(Letter to Professor Potter.)

Zanesville, Ohio, Dec. 1869.

* * * though our Medical Journals have, in part, anticipat-

* Ten minims Liquor Ammonia, twenty distilled water into the radial vein, by Hypodermic Syringe.

ed so much of your paper as relates to Ammonia injection, as a remedy for Snake poisoning; yet as a whole it is interesting. It affords me an opportunity to present you some considerations regarding what is called poisoning, in general, and the mode of operation of organic poisons in particular, from their naked facts; and from a physical basis for the phenomena of organic life.

The foundation has certainly been reached in my division of the forces of organic life into two modes—and these are all the facts justify—viz: organizing, or laborer; and the architect or form giver. The forming, organizing, or laboring force, as common to all organic life; the same in the animal and vegetable realms of nature; and certainly a correlation, or correlations of the ordinary physical forms of nature, viz: light, heat, electricity, etc. The architect of organization, or form force,—that which, from the common physical basis of all life, to wit: albuminous fluids, constructs the various forms of life in the animal and vegetable Kingdoms. In animated nature, the wheaten loaf, certainly is the physical basis for the tissues of all. For of this, man and beast, bird and fish, reptile and insect, may partake, and it does, and will make tissues for all. This is true of the carnivora, as well herbivora Not only so, but most, if not all, the flesh eating animals have the power to assimilate, or change to their forms, the forms or flesh of other animals. Thus, man changes the tissues of the ox to his forms, or the chicken or fish; and vice versa, chicken or fish can return the compliment, and change human flesh into chicken flesh, or forms. Every organic form, therefore represents the combined results of the laborer and architect, just as buildings, bridges, monuments, etc., in the organic world.

Whatever there is then, entitled to the appellation of vital, common to organic life, must be looked for in their forms; for the material is certainly ordinary inorganic matter, and the organization brought about by the ordinary physical forces. The form forces, too, by a little stretch of the imagination, may be said to be common to both organic and inorganic natures; for the definite forms of crystals in the organic world, must be due to a common form force; for nothing, in either organic or inorganic nature, occurs by chance or accident, but in obedience to invariable and inflexible law.

Now what is that we call an organic poison ? and what does it do? and how does it produce the phenomena called "symptoms of poisoning?" How does it produce swelling, paralysis, and death?

The phenomena of organized life, studied on their facts alone, and reduced to their ultimate elements consist simply of form and motion. Bone and brain matter have the same ultimate chemical composition, yet have the most opposite physical and physiological properties, but this difference can be reduced to form and motion in their particles. And what is true of bone and brain matter, is equally true of all other organic tissues.

The factors, then, in ascertaining what an organic poison is, what it does, and how it does it in the living body, are, 1st material; 2nd force; 3rd form; and lastly motion.

An organic poison, in its material, does not differ from the material of the body of the serpent, insect, or other living thing that elaborates it, but it has two other properties, which the tissues and other fluids of serpents do not possess; and the terms which will produce conceptions in the mind, corresponding with the facts, are form and motion. The poison of reptiles, etc., may, also, properly be described as organic germs, and germs having tremendous energy as a form force; and produce effects in the living body in a very different way from potential poisons of the inorganic world, as Prussic Acid, as will be shown hereafter. Reptiles and insects, insert their poison hypodermically; have, in fact, ever since the creation practiced hypodermic injection of their peculiar "Medicine" on their enemies. Since the introduction of the plan of hypodermic medication in our own times, it is now seen that all medical agents act with much greater rapidity in small quantities, than by mouth or rectum.

The phenomena succeeding the hypodermic insertion of the virus of serpents, occur so promptly, that but little time exists for their more careful scientific observation; and the use ot remedies, whether well or ill chosen, soon obscures them. The powerful mental impressions have, also, to be taken into account in the human being. So that to get an understanding of their modus operandi, it is needful to observe the phenomena of other organic poisons, whose effects are more gradually developed, and can, therefore, be more carefully studied. This can be done in small pox and syphillis; and for that matter, in all the so called exanthematous fevers. But take small pox as the example. What does the virus of small pox do in the human system? The simplest, truest and most direct reply is, changes forms. The pock marks are certainly due to changes of form in the skin. And it is not at all likely that these changes of form are confined to the skin, or cutaneous surfaces, seeing that the body, as a living mass of organic matter, is a unity; and whatever changes the forms of the visible parts, it may be safely concluded, modifies, also, the invisible, except, in the instances of mechanical injuries, the action of acids, or alkalies, or heat, or other direct tissue destroying agents. The changes of form, brought about by the small pox virus in the tissues, take place much more slowly than after the hypodermic insertion of the virus of serpents, and can, therefore, be studied more accurately; and because experience has shown that all attempts to arrest its progress are unavailing, it is called a fever, "a burning disease"—has a definite course to run, ending in death or recovery. The tissues of normal forms are decomposed with considerable rapidity. The temperature, as a consequence, ranges much higher than natural. Offensive gaseous odors are given off, as in other instances of the retrocession of complex organic compounds to similar chemical states. Large quantities of solid debris are thrown off from the cutaneous surfaces. The great majority of those who survive these phenomena, enjoy an immunity from a repetition of them through life; for the reason, as it appears to me, that the resulting forms are thereafter in harmony with, or conform to the forms of the form changer, or virus. These phenomena are again illustrated in the so called Scarlatina, where the form of the cutis is so changed as to destroy it, and probably with like changes of form throughout all the tissues. The severe sequalæ so frequently resulting from the so called "Exanthemata," can be understood by referring them to changes of form.

Syphillis, and the so called cutaneous diseases, produce their effects by changing forms. And as the power of remedial agents and measures to restore lost forms are just none at all, they have all along, through the centuries of the past, proved a sore puzzle to the practicing physician. Looking closely into the properties of the most popular remedial agents, they are found among those whose effects are to "promote destructive metamorphosis," or these, combined with agents, to "promote constructive metamorphosis;" with an ample supply of food, rich in the material of tissue. In fact, cutaneous diseases, so called, treated, so as to destroy tissue of lost form and hasten the construction of tissue in its place of normal form, are much more frequently rewarded with successful results, than in any other way. The most successful and popular therapeutic agents for syphillis and cutaneous diseases, are Mercury, Iodine, Bromine, Salines and Caustics, all agents "promoting destructive metamorphosis" of tissue, whether of normal form, or abnormal type; but more particularly of tissue of foreign types or forms.

In your case of Snake bite, the scarifications and escape of blood by the suction of the lips, probably renewed a portion of the organic poison, virus, or form change, of the reptiles. The subsequent violent exercise "promoted destructive metamorphosis," the oxidation and expulsion of the form change; for all work implies waste of tissue. By the increased volume of oxygen introduced into the system at the lungs, in consequence of the exercise, the oxidation and expulsion of the form changing virus, which escaped renewal by the scarification and suction at the point of hypodermic insertion, was certainly hastened.

The success of the whiskey, or spirit treatment, in like cases, probably owes its efficiency to the mode in which spirits influence the organic process of construction and waste of the tissues, which is notably to retard both, thus retarding the process or work of the form change or virus, or it may be due to direct contact with alcohol in the capillaries and circulation; as alcohol enters and finds exit from the system, without undergoing any chemical change, *i. e.*, enters it as alcohol and is expelled as alcohol. But in either way, or by both, time is given to oxidize and expel the intruder or render it harmless.

The changes in the tissues, giving rise to the phenomena of organic poisoning, from the hypodermic insertion of the virus of serpents etc., take place in the molecular structure of the tissues, and the rapidity of molecular metamorphosis, is strongly illustrated by the experiments of Mr. Waterhouse in photography. He calculated that it only required the one twenty-seven thousandth part of a second to fix the solar image on a photographic plate. But this illustration, striking as it is, sinks into comparative insignficance when contrasted with other experiments also, in photography, by Mr. Wheatstone, with the electric spark. On a cylinder he fixed a number of distinct and different pictures. A photograph, by the electric spark, or flash, was taken from this cylinder, while revolving many hundred times per minute, which proved to be, not a confused mingling of the different pictures, but a copy of one picture only. By the aid of a delicate chronoscope, he calculated the duration of the electric spark at not more than one millionth part of a second! And yet in that time, certainly difficult to conceive, if it is at all possible, an image from one of the many pictures on the rapidly revolving cylinder was distinctly impressed on the photographic plate!

It is only with such comparisons as this that the rapidity with which the normal molecular changes in the tissues take place, as well as the development of the phenomena of organic poisoning, after hypodermic insertion, can be comprehended. And the parallel is completely justified by the fact, that the forces in both cases are the same. That is, the forces engaged in the production of a photographic picture, and the phenomena of organic life, in health or disease, are one and the same. The difference in results being due to different modes, or correlations of force. Thus, the force of electricity may be seen at one place depositing, atom by atom, one metal on the surface of another, as in electro-plating; in another instance, it is engaged in transmitting intelligence across continents, and under seas, through thousands of miles of metallic conductors, as in the magnetic telegraph; or in a third, fusing the most refractory metals, as platinum, etc., in the chemical laboratory!

In a recect foreign journal* an account appeared, to the effect, that a house servant of an English surgeon, resident at Inbulpoor, Central India, was bitten by a cobra. The surgeon on being informed of the accident, instantly ordered his carriage. The man's hands were tied to the gig, when he drove the horse steadily a distance of several miles, the man running behind. On arriving at Inbulpoor, the man's body was bathed in the most profuse perspiration, and he was almost exhausted. The further treatment con-

*London Lancet, 1868.

sisted in doses of Eau De Luce and gentle exercise. In a few hours he was out of all danger and recovered.

Dr. Hood, the reporter of the case, in discussing the modus operandi of the virus of serpents, states that Dr. Bence Jones refers the phenomena to the action of a "chemical agent, or poison, producing a mechanical disease." But this formula does not produce mental impression scorresponding with the facts. But by referring the action of a virus to changing form, the mental impression produced, does correspond with the facts, for morbid anatomy and pathogy teaches us, if it teaches us anything, that function depend in form. No organ or tissue would ever fail to perform its function if its normal molecular structure and forms were maintained. A very interesting demonstration of this is found in the case of Mr. Lawler reported by Prof. Bartholow, in the Cincinnati *Lancet and Observer*, for December, last.

But the Indian servant recovered from the effects of the Cobra's form changing virus, and his recovery was one most likely not due to the Eau De Lac, but to his violent muscular exercise, which introduced oxygen in a much larger volume than in a state of repose; if every act of life is at the expense of the tissues; and the presence of oxygen is necessary to envolve the dynamics of organic life. Amidst the greatly increased molecular changes, in consequence of the molecular exercise, the form changing virus was exercised or expelled, or probably both, giving it no time to change forms to such an extent as to destroy the servant's life. In a recent number of an excellent western journal,* a case of Prarie Rattle Snake bite is reported as having been successfully treated by Opium!

Opium is apparently the very opposite of exercise, but on a closer scrutiny it will be seen that its modus operandi must in the end be quite similar to that of spirits, viz: staying the velocity of the processes of construction and waste—constructive and destructive metamorphosis—which stayed the work of the form changing virus, with the result of its oxidation and expulsion, without doing much damage. Then comes your Austrian correspondent, with his injections of Ammonia into the venous system, and even into the heart's centres, with rapid and certain relief! Ammonia is a volatile gas-

---

*Medical Archives, St. Louis, September, 1869.

eous alkali, diffusing itself with great rapidity; .and must influence organic structures in a manner analgous to the fixed alkalies, which are agents promoting the waste of tissue. Thus, salt, (soda and chlorine gas,) used in provisions, in excess, for a length of time, is followed by a scorbutus, useless counteracting agencies are simultaneously made use of, which is beyond all cavil increased waste of tissue. Potassa acts in the same genial way, only less violently, as soda, that is, increases the waste of tissue. The Ammonia, projected directly into the blood stream, would at once retard repair, and greatly accelerate waste, the very conditions brought about by violent muscular exercise; and then meeting the form changing virus in the blood, would probably influence it chemically, so as to materially alter its form changing properties.

In the John Brown case it is stated, consciousness returned at once, after the introduction of Ammonia into the blood stream; the pulse became fuller, pupil responded to light etc. What are these, if they are not dynamics of organic life? And as the dynamics, or forces of organic life are evolved by the oxidation of tissue, or if it is preferred, call it waste of tissue, the Ammonia must certainly have played some part in "promoting destructive metamorphosis," and the resulting organic dynamics of consciousness etc. All these things, designated as poisons, whether organic or inorganic, must act in one of the folloging ways, viz:

1st. Arresting destructive metamorphosis; as by cyanide of potassium, chloroform, prussic accid, etc.

2nd. Promoting destructive metamorphosis; as by strychnia, mercury, etc.

3rd. Diminishing the velocity, or rapidity of tissue metamorphosis; as by alcohol, opium, etc.

4th. Changing forms; as by the virus of serpents, insects, etc., small pox, syphilis, measles. scarlatina, cutaneous diseases, etc.

5th. Immediate destruction of tissue forms; as by acids, alkalies, heat, and caustics.

In investigating the phenomena of organic life, whether in health or disease, it must always be remembered that "for every dynamic result, there must be change of matter." Not only does this law hold good, or intact, in all organic nature, but it is as constant and inexorable in the inorganic world as the law of gravity itself. There is absolutely no exception. The law is as complete, in all respects, as the law of gravity. Has a patient pain? Then the changes of matter are too rapid and out of normal mode. Is he stupid, or sleepy, or comatose? The changes of matter are not proceeding rapidly enough, or in normal mode; or the results of past tissue metamorphosis have not found exit from the body, one or the other, or all three. And these results of the changes of matter swallow up pretty much all the symptomatology of medical literature.

These conceptions of the mode of operation of organic, as well as inorganic poisons—but particularly of the virus of reptiles, insects, etc., and the modus operandi of therapeutic, and remedial agents, and measures for counteracting their effects, in living beings, certainly represent the facts—more difficult to the mind, than they are explained in medical literature elsewhere; even if the molecular changes of metamorphosis, in detail are correctly traced or not. The beginning and end are certainly correctly represented, as well as the visible phenomena between. They have, therefore this value, that the mental impression to which they give rise, correspond with the facts.

Z. C. McELROY.

